# G-Quadruplexes as An Alternative Recognition Element in Disease-Related Target Sensing

**DOI:** 10.3390/molecules24061079

**Published:** 2019-03-19

**Authors:** Jeunice Ida, Soo Khim Chan, Jörn Glökler, Yee Ying Lim, Yee Siew Choong, Theam Soon Lim

**Affiliations:** 1 Institute for Research in Molecular Medicine, Universiti Sains Malaysia, Penang 11800, Malaysia; jeuniceida@gmail.com (J.I.); sookhim89@yahoo.com (S.K.C.); yeeying331@gmail.com (Y.Y.L.); yeesiew@usm.my (Y.S.C.); 2 Division of Molecular Biotechnology and Functional Genomics, Technical University of Applied Sciences Wildau, Hochschulring 1, 15745 Wildau, Germany; gloekler@th-wildau.de; 3 Analytical Biochemistry Research Centre, Universiti Sains Malaysia, Penang 11800, Malaysia

**Keywords:** G-quadruplex, DNAzyme, colorimetric, fluorescence, luminescence, diagnostics

## Abstract

G-quadruplexes are made up of guanine-rich RNA and DNA sequences capable of forming noncanonical nucleic acid secondary structures. The base-specific sterical configuration of G-quadruplexes allows the stacked G-tetrads to bind certain planar molecules like hemin (iron (III)-protoporphyrin IX) to regulate enzymatic-like functions such as peroxidase-mimicking activity, hence the use of the term DNAzyme/RNAzyme. This ability has been widely touted as a suitable substitute to conventional enzymatic reporter systems in diagnostics. This review will provide a brief overview of the G-quadruplex architecture as well as the many forms of reporter systems ranging from absorbance to luminescence readouts in various platforms. Furthermore, some challenges and improvements that have been introduced to improve the application of G-quadruplex in diagnostics will be highlighted. As the field of diagnostics has evolved to apply different detection systems, the need for alternative reporter systems such as G-quadruplexes is also paramount.

## 1. Introduction 

**Figure 1:**
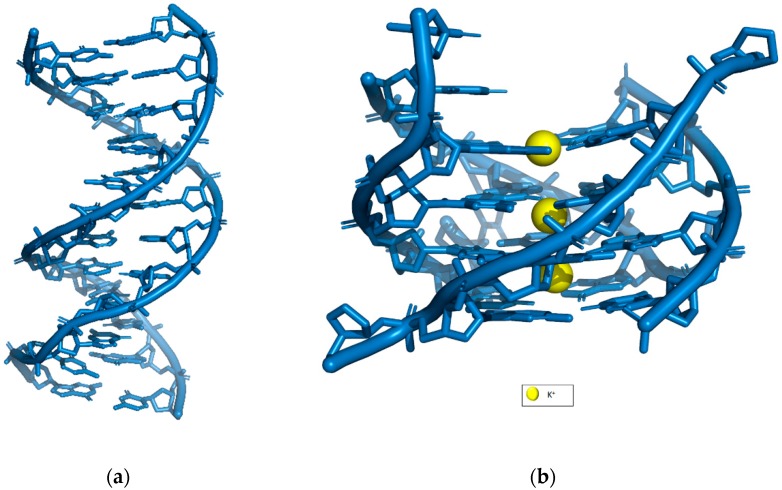
Comparison of (**a**) DNA double helix (PDB: 1BNA) and (**b**) DNA G-quadruplex stabilized by K^+^ (PDB: 244D).

Just over 60 years ago, Watson and Crick published their seminal paper on the DNA structure [1]. In it, they emphasized two principle features of the DNA molecule that are the complementary nature of the base sequences on the two antiparallel oriented strands and the double-helical nature of the polymer. This has helped shaped the way we perceive DNA structure today and allow researchers to design complex nanostructures according to this rule. DNA is a promising engineering material, not just for its outstanding data storage capacity, but for its flexibility, design and efficient chemical synthesis. A deeper understanding of DNA has allowed DNA to expand its presumed main role in genetic-information storage to a more versatile function due to the robustness of the structures it may form. Apart from the Watson-Crick double helix structure, DNA can also adopt alternative structures ranging from disordered single strands to higher-order structures such as G-quadruplex (see Figure 1) as a consequence of dynamic molecular events. 

A DNA G-quadruplex (G4) structure can arise from specific G-rich sequence(s) forming G-tracts. Guanine bases associate with guanines from neighboring G-tracts through Hoogsteen hydrogen bonding to form a square planar called guanine tetrad (G-tetrad) or guanine quartet (G-quartet). The G-tetrad would then stack with one another within the G-tetrad core resulting in a G4 structure.

**Figure 2:**
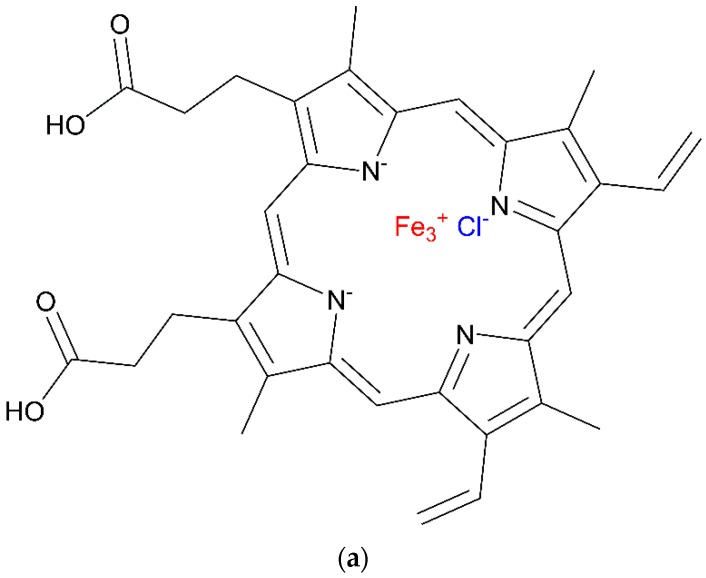

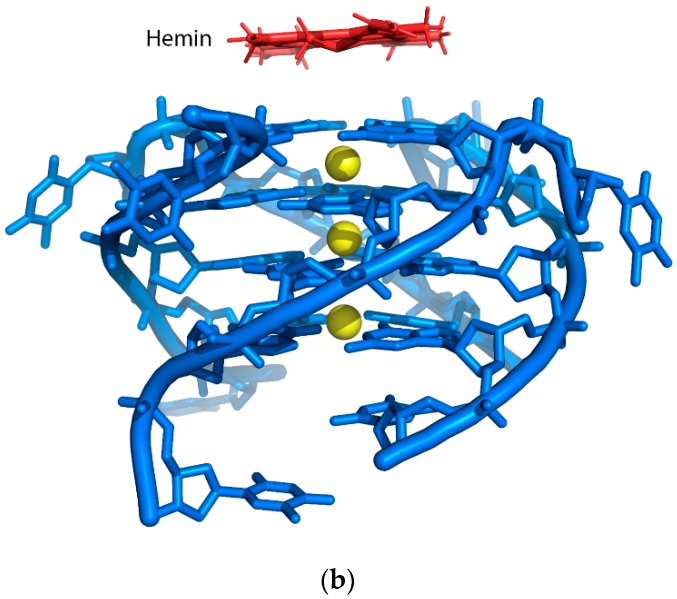
(**a**) Chemical structure of hemin (**b**) G-quadruplex-hemin complex.

Hoogsteen hydrogen bonding, a non-canonical/non-Watson-Crick base configuration allows for the formation of unusual base pairs in certain sequence contexts. G4 arrangements are found to be immensely polymorphic allowing it to represent a huge family of stable structures with a typical overall fold. The rich structural polymorphism motif of the G4 as well as its unique geometry are thought to allow for specific recognition and detection of metal ions, DNA, small molecules, protein and enzyme activity through a number of binding modes in a manner analogous to that of double-helical DNA intercalators [2]. The introduction of G4 structure together with its unique ability to bind with various ligands such as hemin (see Figure 2) have contributed to the identification of new functions of DNA in biology.

Motivated by this fact, the study on DNA G4 has gained attention from different research groups to apply for the design of selective probes in diagnostic assays. This review provides an account of the structural information of G4 besides highlighting its diversity as a reporter in disease-related target detection assays. The assays, specifically targeting DNA, ATP, enzymes and metal ions, are discussed with a focus towards various distinct types of principles and mechanisms that have been performed and reported over the past few years. The claim that G4-based sensors compare favorably with other available detection strategies are discussed as well. It is therefore conceivable that the impact G4 could have on biomedical diagnostic applications will increase with new improvements of the specific G4 design and implementation.

## 2. The Basic Structure of G-Quadruplex

The ability of guanines to self-associate to form four-stranded helical structures, known as G-quadruplex was first demonstrated in 1962 [3]. Even so, the fact that G-rich DNA solutions are able to form gelatinous aggregates was first noted in 1910. However, their exact nature was not noticed until these gels were observed to form stacked planes of guanine tetramers in a cyclic arrangement. Since then, G4 have been extensively studied and were reported to be predominant in functional regions of the human genome and transcriptome. This also includes replication initiation sites, human telomeres, oncogene promoter regions and untranslated regions [4]. The formation of G4 at the telomeres in the macronuclei of the ciliate *Stylonyvhia lemnae* was first visualized using a G4 selective antibody in a biological context [5]. It was suggested based on the telomeric sequence that two overhangs have the propensity to associate end-to-end through the formation of four-stranded conformations of two fold-back hairpins which involves a quadruplex stem. It is further stabilized by stacked layers of G-quartets that are hydrogen-bonded in a cyclic manner [5].

The complementary C-rich strand can adopt another non-canonical structure termed as i-motif. It is based on Hoogsteen C-C^+^ base-pairs, which was initially deemed to form only under non-physiological acidic conditions [6]. However, recently, Zeerati and co-workers discovered that i-motif structures are also able to form in the nuclei of human cells. This is mainly found in regulatory regions of the human genome, which includes the promoter and telomeric regions. In genomic DNA, i-motifs and its complementary G-rich sequences that can adopt G4 structures often coexist. ELISA results shown by this same group proved that iMab recognizes a wide range of i-motif DNA structures with high affinity but it does not bind to G4 structures, suggesting that G4 and i-motif can be well discriminated biochemically [7].

**Figure 3:**
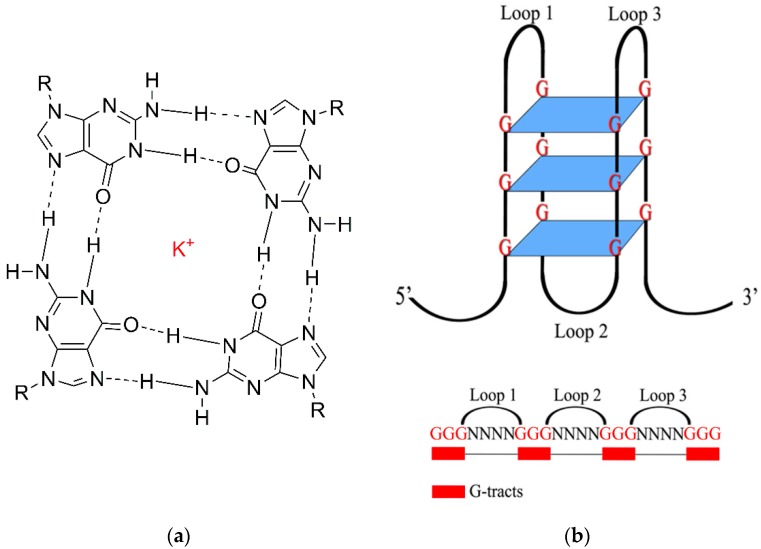
(**a**) Chemical structure of a G-quartet. Four guanines are bonded by Hoogsteen hydrogen bond (dashes) and the monovalent cation K^+^ acts to stabilize the structure. (**b**) An intramolecular G-quadruplex structure consisting of three G-tetrads and a G-quadruplex motif sequence with four G-tracts of three guanines separated by loops.

G4 could be formed from one, two, or four G-rich strands. Although the formation of G4 requires G-rich sequences, not all G-rich sequence can form G4 structures. G4s are composed of at least four G residues as the fundamental building blocks, forming ring-like aromatic planar G-tetrad structures. These structures are connected via Hoogsteen hydrogen bonds, involving the N1, N7, 06 and N2 sites (see Figure 3a) of the guanines [4]. Two or more G-quartets are able to stack on top of one another, resulting in the formation of four-stranded helical structures [8] (see Figure 3b). Loop sequences link the Gs and determine the type of G4 structure to be formed.

Originally, it was assumed that G4s occur only in the form of right-handed helices. However, it was recently discovered that certain G4s such as the therapeutically active G-rich oligonucleotide AGRO100 (also known as AS1411) adopts a left-handed helical conformation [9]. Thus, aside from the Z-form of double-stranded DNA (dsDNA), G4s can also adopt the unusual left-handed helix. This property is critical if more specific ligands are to be designed that can distinguish between the helical conformations.

Various rules governing G4 folding have been established with the ongoing advancement in the structural studies of G4 [10,11,12,13,14]. The folding of G4 can occur through the folding of a single strand (intramolecular) or the association of two (bimolecular) or four (tetramolecular) separate strands (intermolecular) [12]. Intramolecular interactions in single-stranded DNA connects three loop regions linking four G-tracts of at least two consecutive Gs together [13].

**Figure 4:**
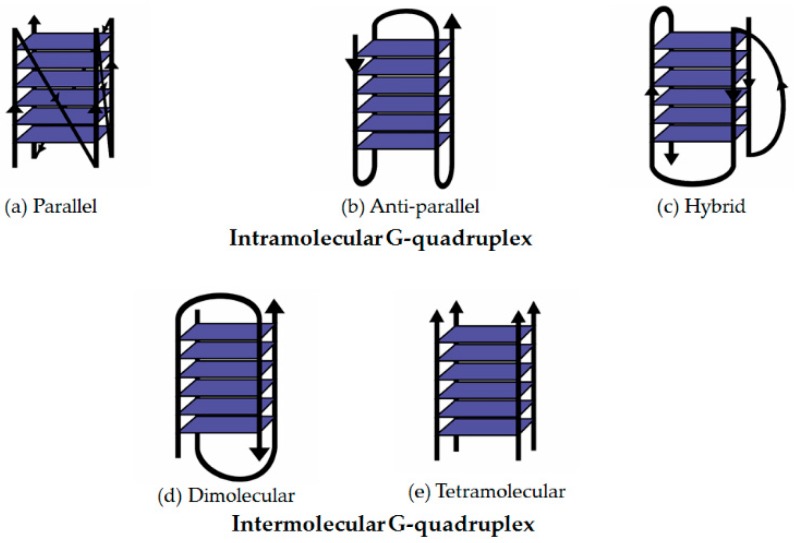
Common conformations of G-quadruplex. (**a**) Parallel (**b**) Anti-parallel (**c**) Hybrid (**d**) Anti-parallel (dimolecular) (**e**) Parallel (tetramolecular).

Different sequences will evidently adopt alternative topologies, but in some cases various conformations may also be derived from one particular sequence [14]. Depending on the environmental conditions, the conformations can interchange or are found to co-exist in different states in parallel. Thus, it is not uncommon for quadruplex structures obtained by crystallography to disagree with those obtained in solution [15]. Each of the four G-tracts can run in similar (parallel) or different (anti-parallel) direction with respect to each other [4,16]. Anti-parallel conformation may be classified into two types as the guanines involved have alternating *syn-* and *anti*-glycosyl conformation along each strand [17]. In addition, G4 have also been observed to adopt mixed orientations consisting of parallel-antiparallel strands to form a stable structure [18]. This hybrid orientation of G4 contains both consecutive guanines in the same (*syn-syn* or *anti-anti*) and different (*syn-anti* or *anti-syn*) conformations [17]. The four G-tracts are comprised of the same guanine rich oligonucleotides connected by loops of variable nucleotide lengths and sequences [16]. Figure 4 highlights some of the basic types of G4 conformations: parallel, antiparallel and hybrid (see Figure 4).

The folding of G4 occurs rapidly and the obtained structures are complex, exhibiting great conformational diversity [14]. This structural polymorphism is mainly due to the properties of the loop, which includes strand polarity, variations in strand stoichiometry and the sites on the loop that are connected to the G strands [4,19]. The structure of G4 may also depend on the length and composition of DNA or RNA, the positions of the loops, the orientation of the chain and the nature of the cations involved [16]. G-quartet regions, groove dimensions, central channel of the G4 as well as the negative electrostatic potential of the anionic backbone are factors that influences the binding selectivity of the target molecules [4].

Other than the sequence requirements, the integration of cations with the G4 is also vital in determining the polymorphism and stability of the G4. The ligands act to coordinate with four electronegative O6 atoms from a G-tetrad and another four of the adjacent stacked G-tetrad. In the presence of different cations, the G-rich sequence may adopt different structures [8,14]. This occurs as such ions contribute to variation in the interaction with the grooves and loops [20]. The quartet arrangement generates a central channel that allows the cations to settle which influences the interaction of the G4 to the cations. Depending on the type of cations and the specific structure of the G4, the positions of the cations can be located along or between the quartet plane [20]. Important features of the cations and their structural influence are their radius and valency. Cations supporting the G4 structure are usually metal ions with the notable exception of the monovalent ammonium ion [21]

**Figure 5:**
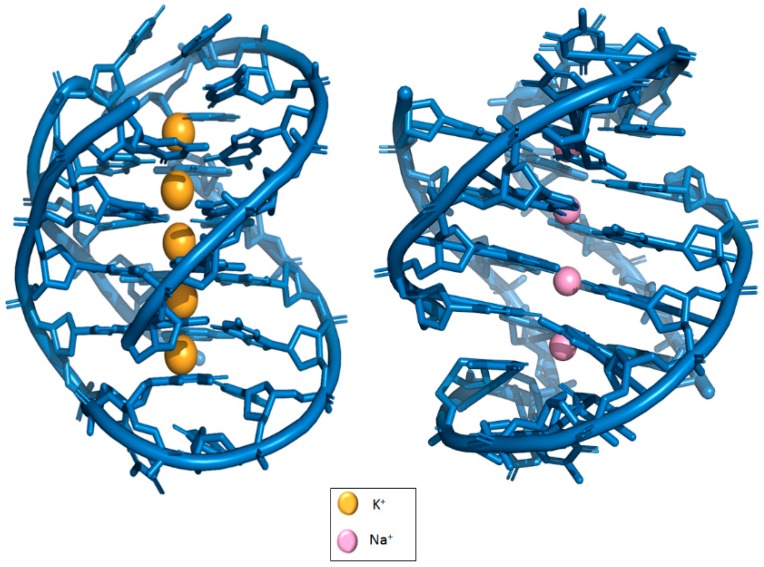
Location of K^+^ (PDB: IJPG) and Na^+^ (PDB: IJB7) in G-quadruplexes.

Generally, G4 formation and stabilization require monovalent cations, in particular, Na^+^ and more commonly, K^+^. K^+^ is claimed to be more biologically relevant compared to that of Na^+^ due to its higher intracellular concentration. The nature of the ions affects their exact location between tetrads. K^+^ are observed to be *ad libitum* equidistant between each tetrad whereas Na^+^ are located within a range of geometries of a G4 [14]. Na^+^ ions are slightly displaced from the G-quartet due to the electrostatic repulsion between adjacent Na^+^, resulting in a slightly altered coordination geometry (see Figure 5) [22]. Not only K^+^ has a better coordination with 06 guanines, this cation also possesses lower dehydration energy. In Na^+^-containing solutions devoid of K^+^, G4 formation can also occur but at a much slower rate [23]. Therefore, K^+^ in general is preferred over Na^+^ in G4 formation. Another study conducted also showed in the presence of KCl, the activity doubled that of NaCl [24].

It has also been reported that other monovalent and divalent ions can in fact affect the stability and structure of G4 [20]. It was suggested that the stability of G4 structures is influenced by ions in the order of K^+^ > Ca^2+^ > Na^+^ > Mg^2+^ > Li^+^ and K^+^ > Rb^+^ > Cs^+^ [25]. It was also demonstrated that interaction of G4 with type Ia and IIaa cations with respect to the stabilization of G4, follows the order of Sr^2+^ > Ba^2^+ > Ca^2+^ > Mg^2+^ and K^+^ > Rb^+^ > Na^+^ > Li^+^ = Cs^+^ [26]. In another study, silver ions (Ag^+^) was shown to also coordinate with guanine bases [27] and disrupt the structure of G4 significantly [28]. This happens as Ag^+^ chelates guanines at the binding sites, N_7_ and C_6_O [27], which are involved in G4 formation. This results in the inhibition of the catalytic activity exhibited by G4-hemin DNAzyme [28]. A further study has shown that no high order structural change was observe upon Ag^+^ addition, suggesting the destruction of G4 structure [29].

Disruption of G4 structures have also been observed with some divalent cations. Divalent cations such as Mn^2+^, Co^2+^ or Ni^2+^ may disrupt the structure of G4 even in the presence of K^+^. Divalent cations are capable of destabilizing G4 when the G4 and monovalent cation concentrations are sufficiently low [30,31]. However, the effect of trivalent metal ions on G4 structures has not been well studied yet. Nevertheless, the trivalent ions were observed to destabilize G4 structures, as they were found to be remarkably reduced in the presence of metal chelators [32]. It was also found that the stacking of the quartets is better promoted in the presence of trivalent lanthanide metal ions such as La^3+^, Eu^3+^, Tb^3+^, Dy^3+^ and Tm^3+^ [33]. This information is critical in the design of stable G4 structures for various applications.

**Figure 6:**
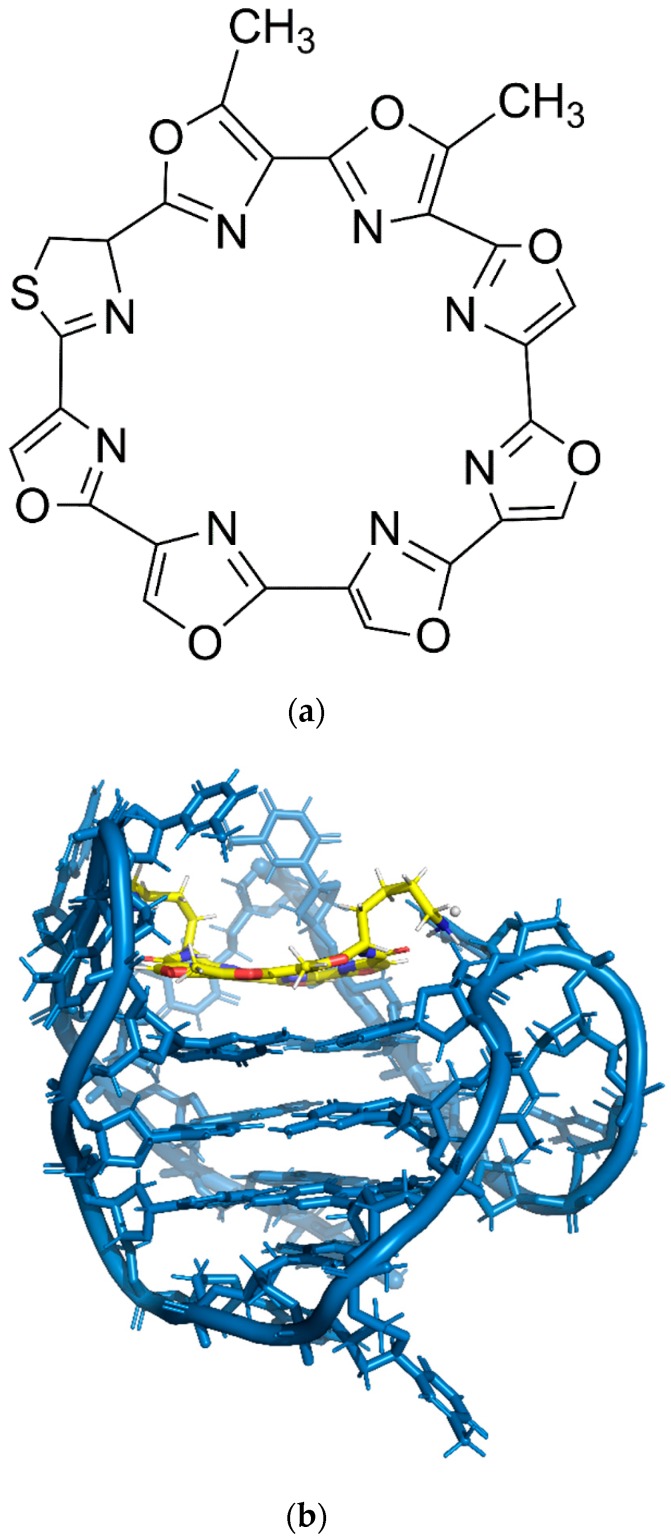
(**a**) Chemical structure of telomestatin. (**b**) Solution structure of an intramolecular (3 + 1) human telomeric G-quadruplex bound to a telomestatin derivative (PDB: 2MB3).

**Figure 7:**
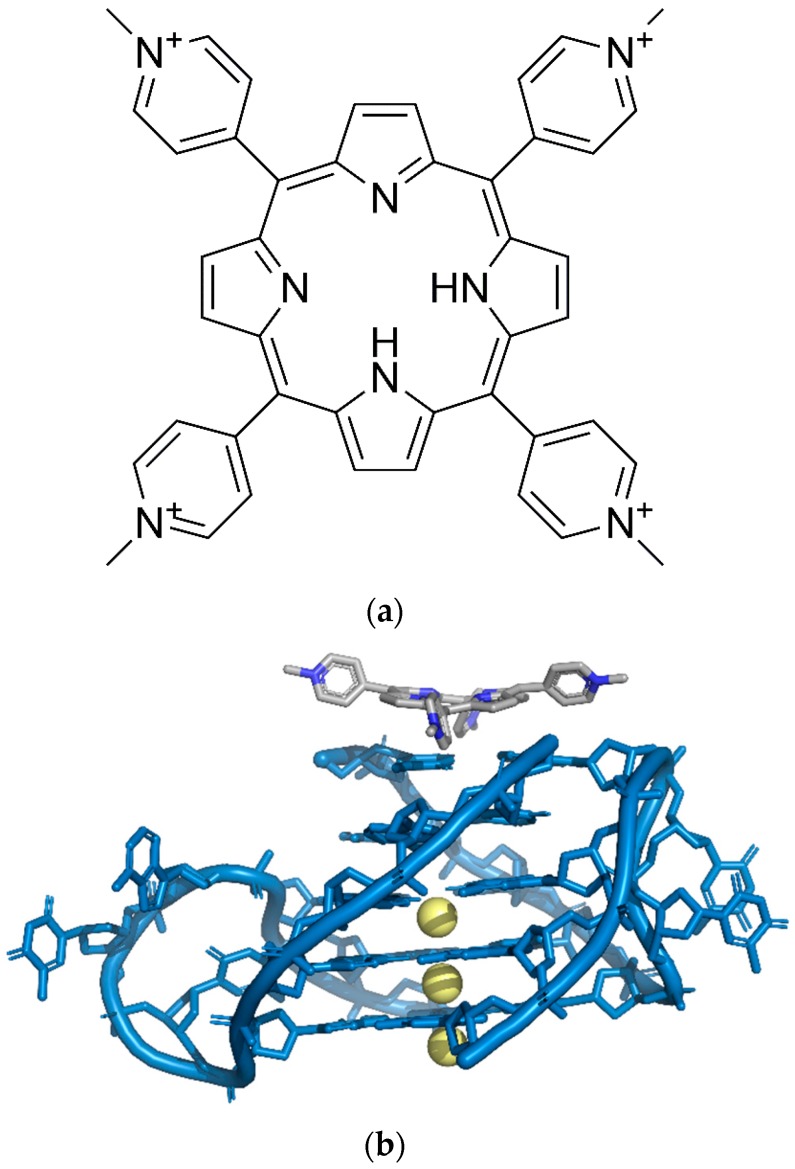
(**a**) Chemical structure of TMPyP4 (**b**) A parallel stranded human telomeric quadruplex in complex with the porphyrin TMPyP4 (PDB: 2HRI).

The effect of a number of G4-binding ligands on G4 structure and stability have been widely studied. Telomestatin, a natural product isolated from *Streptomyces anulatus* 3533-SV4 has been shown to be a very potent telomerase inhibitor. This is suggested by the similarity of the structure between telomestatin (see Figure 6a) and G-tetrad. The ability of telomestatin to either trap out pre-formed G4 structures or facilitate the formation of G4 (see Figure 6b) can result in the sequestering of the telomeric sequence (single-stranded d[T_2_AG_3_]_4_ sequencecrucial for telomerase activity). Higher concentrations of telomestatin has been found to cause a significant increment of intramolecular basket-type G4, suggesting that the telomestatin stabilizes or induces the intramolecular G4 formation [34]. Interestingly, telomestatin can replace the need for monovalent cations, specifically K^+^ or Na^+^ to stabilize intramolecular G4 structure, which is a unique trait among G4-interactive compounds. In contrast, a porphyrin compound, 5,10,15,20-tetrakis-(*N*-methyl-4-pyridyl)porphine (TMPyP4, see Figure 7a) has been shown to preferentially facilitate the formation of intermolecular G4 structure (see Figure 7b). TMPyP4 is responsible to suppress the proliferation of alternative lengthening of telomeres in (ALT)-positive cells and inducing anaphase bridges in sea urchin embryos. However, telomestatin does not possess this effect suggesting the selectivity of telomestatin for intramolecular G4 structures and TMPyP4 for intermolecular G4 structures [35]. TMPyP4, mainly known as DNA G4 stabilizer, was also reported to be able to unfold an extremely stable all purine RNA G4 (M3Q) and regulate the activity of a reporter gene through the disruption of G4 [36].

**Figure 8:**
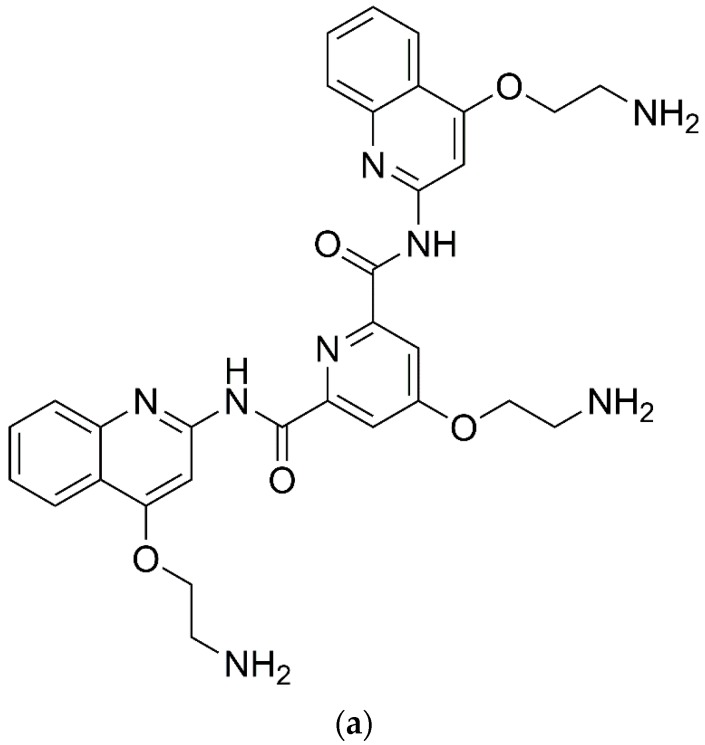

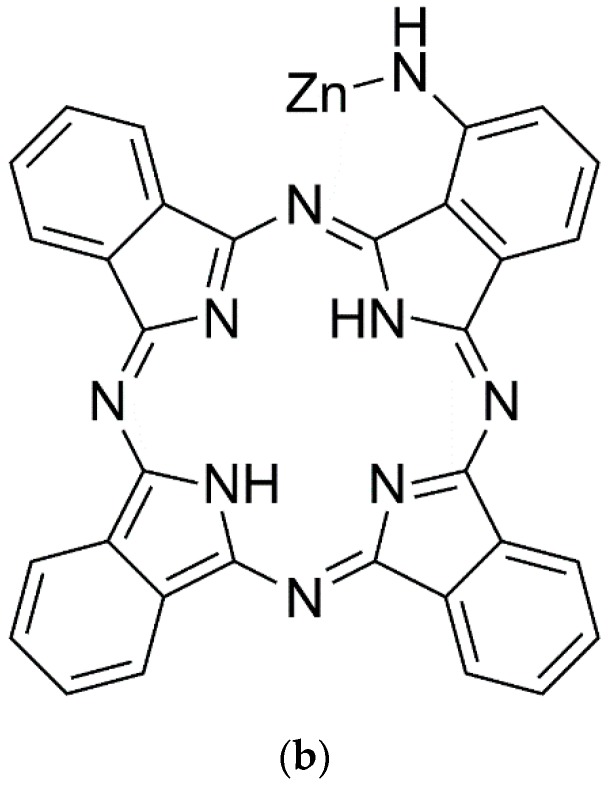
Chemical structure of (**a**) pyridostatin and (**b**) zinc aminophthalocyanine (ZnAPC).

Pyridostatin (see Figure 8a) is a small molecule that is known to particularly stabilize G4 DNA complexes, triggers neurotoxicity and induce DNA double-strand breaks (DSBs) in cultured neurons [37]. Also, zinc aminophthalocyanine (ZnAPC) (see Figure 8b) has been demonstrated to bind to a G4-forming oligonucleotide derived from 5′-untranslated region of *NRAS* mRNA, causing a selective cleavage of the target RNA G4 upon photo-irradiation [38].

Pressure is a key thermodynamic factor that can induce structural changes in biomolecules due to a volumetric decrease [39]. Depending on the specific sequence, G4 structures have been found to exhibit exceptionally high thermodynamic stability [40]. This superior property has been exploited in applications such as isothermal amplification in order to disrupt the duplex structure for primer binding [41]. However, non-canonical DNA structures are more sensitive to the effects of pressure compared to duplexes which have higher stability under pressure [39]. G4 exhibits positive and large Δ*V*_tr_ values, suggesting that thermal stability would decrease with increasing pressure resulting in the unfolding of G4. A study conducted using UV melting under high pressure was able to monitor the unfolding process of human telomeric oligonucleotides. It was suggested that the ΔT_m_/ΔP is less than −10 × 10^−2^ K·MPa^−1^. Hydration of biomolecules is largely affected by pressure, unlike folding of a nucleic acid duplex which takes up water molecules during the folding process. Osmotic pressure analysis showed that water molecules are released during the folding of G4 [39]. Therefore, there is a potential to develop nano-materials triggered by pressure effects, considering the difference in sensitivity of each DNA structure to pressure.

Previously available assay allows the identification of G4 formation with hyperchromism at 260 nm and hypochromism at 295 nm but it is not able to identify all three (parallel, antiparallel and hybrid) G4 types [42]. A recent study investigated the relationship between thermal denaturation profiles and G4 structure based on UV absorbance [17]. The melting profiles of three types of G4 structures (parallel, antiparallel and hybrid) at 243, 260, 275 and 295 nm suggest that the G4 topology affect the denaturation profile of G4 (see Table 1). The work also developed an inexpensive assay that not only distinguishes G4 and DNA duplexes or triplexes, but also between parallel conformation of G4 with the other two structures (antiparallel and hybrid). This was achieved due to the hypochromism exhibited by parallel G4 structures in contrast to the antiparallel and hybrid structures which shows hyperchromism at 275 nm [17]. These recent findings on thermal denaturation profiles of G4 could potentially boost further development of G4 in sensing systems and provide new insights for the establishment of novel sensing strategies.

## 3. G-Quadruplex-Based Detection System

### 3.1. DNAzyme-Based Colorimetric G-Quadruplex/Hemin System

#### 3.1.1. Conventional DNAzyme-Based G-Quadruplex/Hemin System

**Figure 9:**
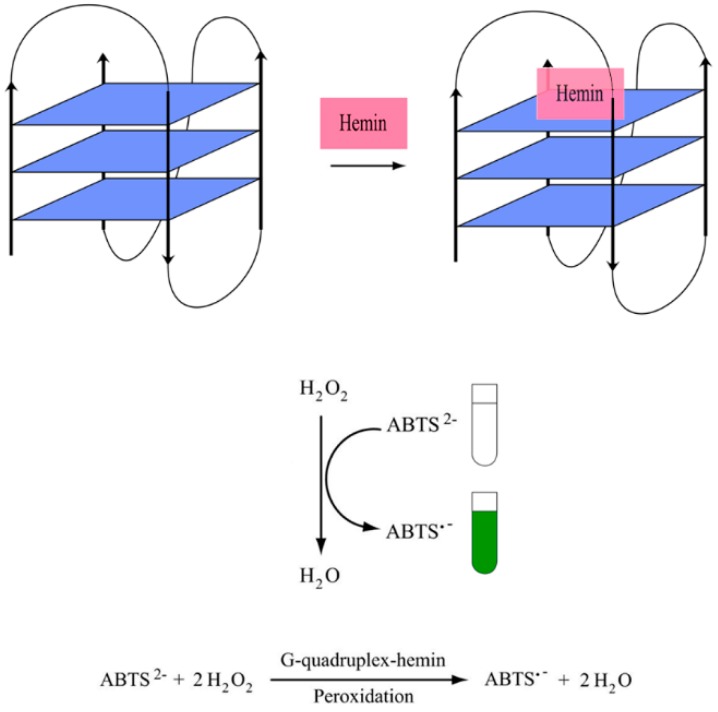
A schematic illustration of simple colorimetric G-quadruplex DNAzyme system.

Horseradish peroxidase (HRP) can be used in an enzyme-conjugated assay as a label mainly to detect proteins in immunoassays and can also be adopted for DNA detection too. As modifications can be expensive and cumbersome, the availability of a peroxidase-mimicking DNAzyme formed by the binding of a G4 with hemin is an interesting alternative [43,44,45]. The peroxidase mimicking catalytic activity of G4 DNAzyme catalyzes the oxidation of 2,2′-azinobis(3-ethylbenzothiazoline-6-sulfonic acid) (ABTS^2−^) to produce a colorimetric change from a colorless solution to a visible blue-green product (see Figure 9). DNAzyme systems involving other substrates like luminol, 3,3′,5,5′-tetramethylbenzidine sulfate (TMB), 4-chloro-1-naphthol (4-CN) or scopoletin (Sc), in the presence of hydrogen peroxide (H_2_O_2_) can also be used to produce colorimetric, electrochemical and fluorescence readouts [46,47,48,49,50,51]. However, colorimetric G4-hemin DNAzyme-based biosensors are utilized in diagnostics for its simplicity, fast analysis, low cost and most notably, the ability to detect target molecules visually. The ABTS^2−^ substrate supplemented with H_2_O_2_ DNAzyme can be designed to generate the colorimetric change as the outcome of the detection of a particular target.

**Figure 10:**
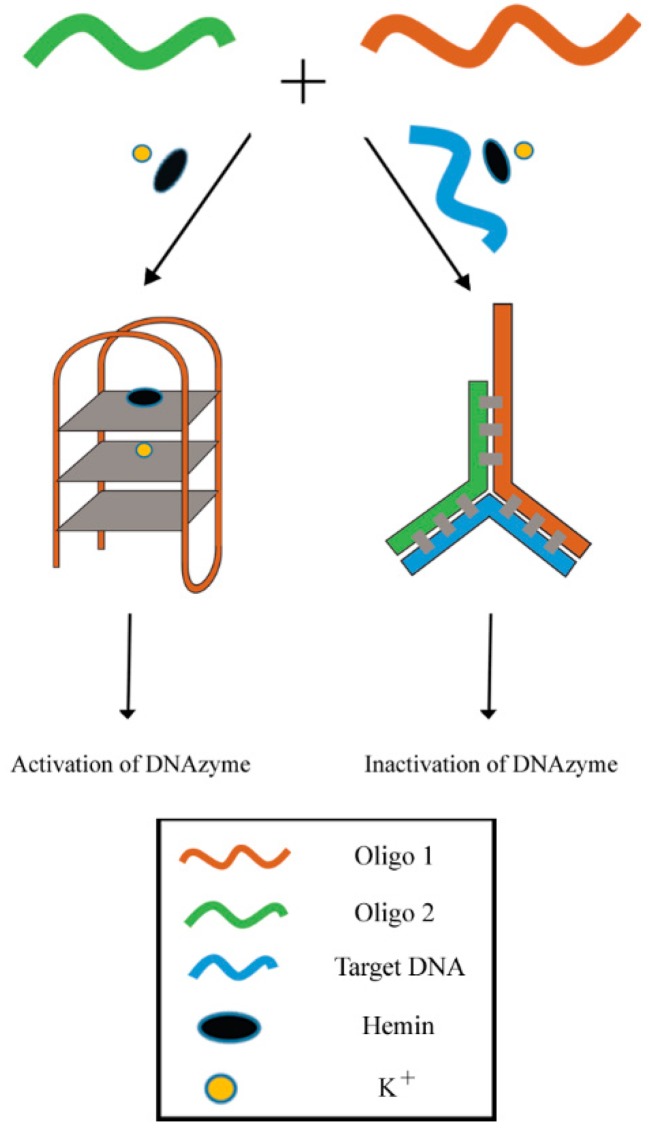
Schematic illustration for sequence-specific recognition of single-stranded DNA based upon Y-shaped DNA duplex and G4-hemin DNAzyme.

Single nucleotide polymorphisms (SNPs) is a condition that may lead to the development of numerous human diseases which includes complications such as Alzheimer’s, sickle cell anemia, cystic fibrosis and certain cancers [52]. A series of sensors utilizing protein enzymes have been established for the detection of SNPs. Restriction enzymes can also be employed to detect SNPs, but are limited to the recognition sequence of the enzyme recognition domain. Ligase was also reported to be capable of detecting SNPs in random DNA sequences [53,54], but this type of assay is relatively expensive and vulnerable to the surrounding conditions [55]. In view of the limitations of existing methods, a turn-off colorimetric sensor was developed utilizing the aforementioned DNAzyme system for the recognition of SNPs based on G4-hemin DNAzyme and Y-shaped DNA duplex. In this assay (see Figure 10), Oligo 1 (G-rich sequence) and Oligo 2 were designed to be partially complementary to each other. Oligo 1, Oligo 2 and target DNA will hybridize with each other to form a Y-shaped DNA duplex (3-way junction) in the presence of target DNA. This will render the DNAzyme sequence unavailable for catalytic reaction, thus inactivating the DNAzyme. Therefore the resulting outcome is a decrease of the absorbance readout of the oxidation product of ABTS^2−^ [56].

An abnormal concentration of sodium (Na^+^) will affect both cellular and electrical functions in biological systems. Hence, it is associated with many diseases such as heart failure and kidney diseases. Quantitative detection of Na^+^ is required for the diagnosis of such diseases. However, a large amount of K^+^ can interfere with Na^+^ sensing in physiological systems making the development of Na^+^ detection systems a huge challenge. As G4s have a higher sensitivity towards K^+^ compared to Na^+^, this makes G4 based sensor development for Na^+^ even more challenging. A study reported the development of a Na^+^ ion sensor guided by the fact that there are specific G4s possessing different conformations depending on the presence of either K^+^ or Na^+^ [57]. In this study, a known G4 named p25 was utilized due to its ability to exhibit an antiparallel conformation with Na^+^ but a hybrid conformation upon the presence of K^+^. With increasing Na^+^ concentration, even in the presence of K^+^, the p25 G4 undergoes a specific conformational transition. Due to the difference in binding affinity of G4 various conformation towards hemin (discrepancy in catalytic activity), and by applying the ABTS-H_2_O_2_ system, a colorimetric probe for Na^+^ was successfully established [58].

Further research on G4-hemin DNAzyme reported that terbium/G4-hemin DNAzyme is able to exhibit a significantly higher catalytic activity in comparison with K^+^-induced G4-hemin DNAzyme [51]. The quantitative data obtained from this study showed that the activity factor of Tb^3+^-induced G4-hemin DNAzyme and K^+^-induced G4-hemin DNAzyme were 11.55 and 2.02 respectively [51], clearly indicating that the former has a higher peroxidase activity than the latter. This would allow for a wider application of the sensing platform as it was previously thought to be restricted to only K^+^ and Na^+^ as the inducer of G4 structure formation.

It was mentioned earlier that G4-hemin DNAzyme would be significantly inhibited in the presence of silver ions [28]. However, the addition of hydrogen sulfide (H_2_S) is able to remove the coordinated Ag^+^ through competitive binding owing to its strong affinity to Ag^+^ [59]. This would then remove the inhibitory effect by Ag^+^, thus by reforming the G4 as well as its peroxidase-like activity. CD spectra analysis of Tb-induced G4-hemin DNAzyme showed a structural change to the original state upon addition of H_2_S in the presence of Ag^+^ ions. The ability of Tb^3+^-induced G4-hemin DNAzyme to exhibit a higher catalytic activity compared to K^+^-induced G4-hemin DNAzyme, a colorimetric detection system for H_2_S was developed [29]. This is important as H_2_S is found to take part in various physiological processes and its abnormal level has been associated with symptoms of various diseases such as Down syndrome, Alzheimer disease and diabetes. In this setup, Tb/G4-hemin DNAzyme was first prepared before introducing Ag^+^ to suppress the peroxidase-like activity. The addition of Ag^+^ causes the green color of the solution to change to colorless and the absorbance of ABTS-H_2_O_2_ to decrease, reflecting the loss in catalytic activity. A green color was found to reappear upon the addition of H_2_S indicating the reformation of the G4 structure and the recovery of the peroxidase-like activity. With the enhancement of H_2_S concentration, the absorbance of ABTS-H_2_O_2_ increases gradually which forms the basis of the system. The selectivity of this detection system was examined by introducing various competing anions. Even at concentrations of 100-fold higher, the tested anions were not able to recover the catalytic activity of Tb/G4-hemin DNAzyme. This implies the sensitivity of the detection system towards H_2_S [29]. The claim that Tb^3+^-induced G4-hemin DNAzyme exhibits a higher peroxidase-like activity than the G4-hemin DNAzyme induced by K^+^ and Na^+^ was further examined in this study by adding K^+^ and Na^+^ into the system instead of Tb^3+^. The result was found to be consistent in which the Tb^3+^-induced system was able to exhibit higher peroxidase-like activity [51]. Not only did this system exhibited a higher catalytic activity, it also displayed the ability to be applied on serum samples with no retardation of activity [29].

#### 3.1.2. DNAzyme-Based Colorimetric Split G-Quadruplex System

It was found that the structure of G-rich nucleic acid sequences can be split to two parts and act as a binary probe, which are reported to exhibit extraordinary selectivity [60]. In a split system, two separate short G-rich oligonucleotides can be combined to form a common G4 wherein the guanines of the G4 are usually distributed on two different strands allowing the target strand to bring them together through hybridization. Formation of a G4 structure can thus turn on the catalytic property for signal readout [61]. The number of bases of the whole split G4 is often twelve. In has been reported that the separation of split G4 designs was best implemented in the loop region of the G4, thus the twelve G bases of the G4 are always divided in ratios of either 2:2 or 1:3. However, a study reported that the assembly of two equally-split parts could easily form an active hemin aptamer even in the absence of target DNA producing a background signal [62].

Hence, a number of studies resorted to splitting the unabridged G-rich strand into an asymmetric ratio instead [63,64]. In a system involving the asymmetrical split DNAzyme strategy to rapidly and visibly detect rifampin-resistant *M.tb* with mutations in the *rpoB* gene, unequal ratio (3:1) of probe A and probe B were assembled through hybridization with the target ssDNA [64]. Since the target DNA was originally in dsDNA form, asymmetric PCR was applied to produce ssDNA. To ensure sufficient quantity of target DNA was available and to increase the sensitivity of the assay, nested PCR was conducted. Interestingly, the PCR product was added into the detection system without the need for purification prior to the assay. A new strategy was designed to minimize the adverse effects of complicated secondary structures of the target DNA to further improve the efficiency of the probes. The 5′-end of Probe A was designed to match the 3′-end of the target ssDNA with three GGG repeats. The 3′-end of Probe B contained one GGG repeat was designed to match the 5′-end of the target DNA. Therefore, two dsDNA were formed at the two ends of the target DNA when the target DNA was added to the mixture of probes A and B. A G4 structure was formed in the middle due to the folding of the overhanging strands from Probe A and Probe B. The formed DNA G4 is then converted into a DNAzyme that will subsequently catalyze the H_2_O_2_-mediated oxidation of ABTS^2−^ through peroxidation-like activity when bound with hemin. As a result, an oxidation product, ABTS^−^ was formed and a visible green color was observed [64].

Further studies on split G4 have been done to elucidate the effects of split G4 guanine ratio. The effects of separation between the guanine bases with either more or less guanine bases in different strands of the split G4 was studied to determine the optimal ratio to split the G4. Six split modes were tested and it was shown that the performances of the modes 3:9 and 6:6 were both moderate whereas the best performing mode was 4:8 [65]. The employment of split G4 has allowed an additional dimension in sensor designs affording more flexibility. In comparison with G4 detection systems using unabridged probe strands where the results are limited, this splitting strategy allows the G-rich segments to integrate into many patterns and the best ratio mode could be identified, leading to a reduced background signal.

#### 3.1.3. DNAzyme-Based Colorimetric G-Quadruplex/Hemin Molecular Beacon System

**Figure 11:**
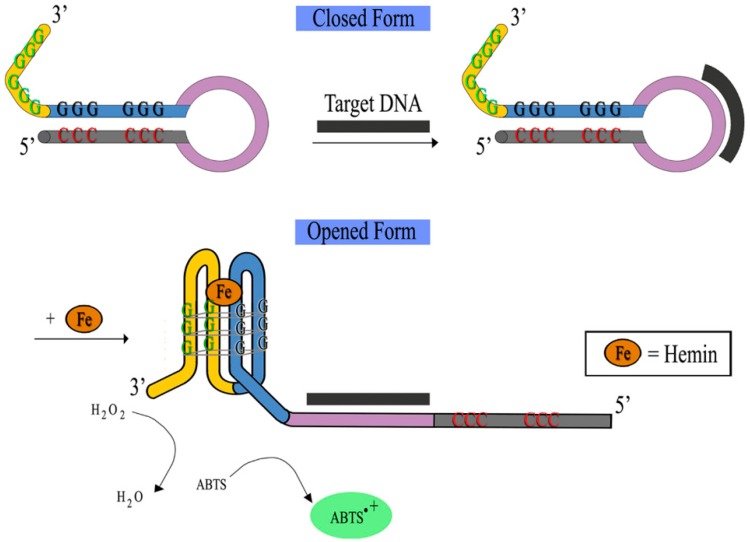
Typical DNAzyme-based colorimetric G-quadruplex/hemin-molecular beacon system.

The split G4 design can also be applied to the molecular beacon (MB) concept devoid of the fluorophore-quencher complex whilst maintaining their specific catalytic activity. MB was first reported in 1996 and has since been one of the most widely used systems for DNA biosensors [66,67]. Originally, the MB system alone relies on the concept of a closed to opened conformational change in the presence of a target DNA. The conformational change can be detected with simultaneous production of a fluorescence signal. MBs are basically hairpin-shaped oligonucleotides that contain a stem-loop structure [68]. The DNA bases on both sides of the stems are connected by hydrogen bonds whereas the 5′- and 3′-end of the MBs are respectively labeled with a fluorophore and a quencher [69]. The construct will then unfold in the presence of the target sequence to hybridize, resulting in the separation of the fluorophore from the quencher to yield a fluorescent output. However, besides being costly it is known that the utilization of the fluorophore and quencher also requires complicated modification and complex operation making it cumbersome [69]. In this alternate rendition of MB, G4 MB-based detection of nucleic acids involves no fluorophore-quencher complex but incorporates the guanines needed for the formation of the G4 into the stem formation of the MB (see Figure 11). Subsequent binding to the target will open the MB stem permitting the guanines to be made available to engage in G4 formation.

Considering the advantages of MB and split G4 with the drawbacks of employing the fluorophore and quencher, a study combining the MB technique and split G4 structure to produce a label-free G-quadruplex DNAzyme MB oligonucleotide sensor for *Pseudostellaria heterophylla* (PH) was developed [70]. In this system, a split mode G4 was utilized in place of the fluorophore/quencher combination where one GGG repeat served as a reporter and tethered to both ends of the MB structure. A section of the oligonucleotide specific for the target sequence was made to complement the loop portion and act as the sensing element of the assay [70]. In the absence of target DNA, one pair of GGG repeat associates and forms an intermolecular G4 structure. A DNAzyme is generated through the high affinity binding of hemin to the formed G4. This complex results in a peroxidase-like activity to produce a colored product with a strong UV-vis absorption signal. In the presence of the target DNA, the target DNA will hybridize to the loop of the MB inhibiting the formation of intermolecular G4 DNAzyme due to the opening of the stem to form an inactive duplex DNA structure. This results in a catalytic activity loss with a weaker UV-vis absorption signal in the presence of the target DNA [70].

Not only can a DNAzyme MB act as a catalytic enzyme and target recognition element, this multifunctional label-free probe can also function as a primer for polymerization [71]. A novel label-free DNAzyme MB strategy was developed for the colorimetric amplification detection of p53 DNA. For the DNAzyme MB probe alone, in the presence of target p53 DNA the catalytic activity of peroxidase-mimicking DNAzyme is locked in its hairpin structure resulting in a signal readout. Meanwhile, the hybridization of the recognition element of MB with target DNA promotes the formation of G4, triggering isothermal circular strand-displacement polymerization (ICSDP) reaction. This provides a positive readout signal and dynamic response range of 7 orders of magnitude even without chemical modifications and additional nucleic acids [71].

### 3.2. Non-Covalent Fluorescence-Based G-Quadruplex System

#### 3.2.1. Conventional Non-Covalent Fluorescence-Based G-Quadruplex Sensing System

The early studies in the field of DNA-based sensing commonly used covalently-conjugated oligonucleotides which are labeled with fluorophore/quencher or donor/acceptor pairs. However, covalent conjugation of the fluorophore has some drawbacks as it may interfere with the operation of the assay, influencing the selectivity and binding affinity of the functional oligonucleotides. Another limitation is the cost and time required to produce such conjugated oligonucleotides. Thus, label-free approaches have emerged as a practical and cost-effective alternative to the fluorescently-labeled oligonucleotides in DNA-based sensing applications. This alternative approach allows for the probes to interact non-covalently with DNA via different binding modes and eliminates the need for covalent attachment to the nucleic acid backbone. The probes may bind either through intercalation, groove binding, end stacking or electrostatic interactions that is designed not to affect the functionality of the DNA [72,73].

**Figure 12:**
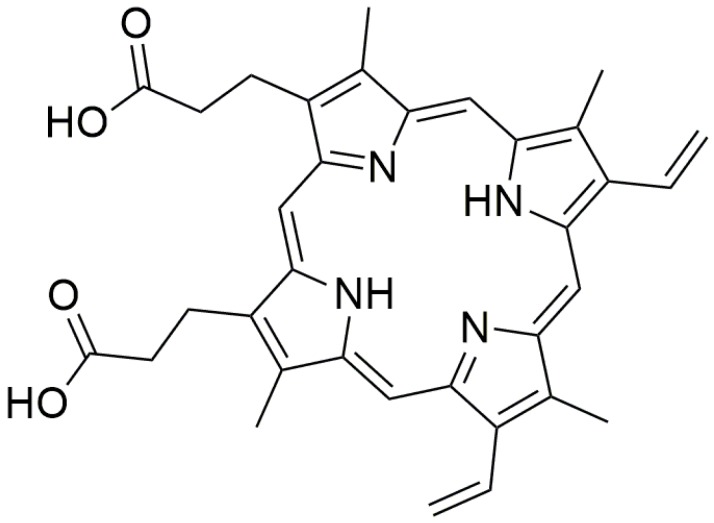
Chemical structure of protoporphyrin IX (PPIX).

Commonly used fluorescent G4 dyes includes derivatives of carbocyanine, phthalocyanines, porphyrin, carbazole, anthracyclines, ethidium bromide and triphenylmethane [73,74,75]. One of the most popular and commonly studied G4 ligands which binds and stabilizes different types of G4 structures is porphyrin [74]. Their binding to G4 can dramatically enhance the florescence intensity of porphyrins. Protoporphyrin IX (PPIX) (see Figure 12), a ubiquitous heme precursor, has been shown to be a G4-selective fluorescent probe in vitro. Fluorescence enhancement was reported to increase by 16-fold with the addition of G4 to PPIX [76]. The preference of using PPIX as a signal reporter is mainly due to the large dependency of the affinity between it and the G4 on the integrity of G4 [77]. It exhibits weaker affinity for duplex and anti-parallel G4 DNA, but selectively binds to parallel G4 DNA. In addition, the affinity can be controlled by the input DNA through hybridization [74,78].

**Figure 13:**
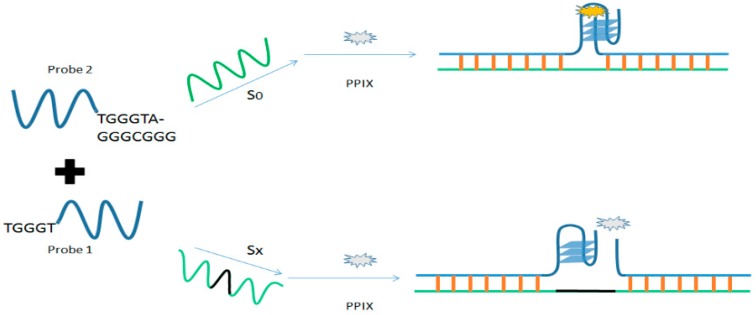
Activation of fluorescence of PPIX triggered by distance between two split G-quadruplex strands (x: number of bases added).

In a study conducted to measure the length of DNA based on the base number between two selected sequences, PPIX was chosen as a probe to study the split G4 formation (see Figure 13). Therein, two G-rich strands were hybridized to the target strand, driving the G-rich strands together in the middle of the complex molecule. PPIX in solution bound to the G4 and its fluorescence intensity decreased dramatically in the presence of additional bases in the middle of the target strand. The addition of extra bases in the middle of the target strand results in an unstable structure of split G4 due to the increase distance between them. This in turn decreases the affinity of the split G4 for PPIX resulting in a decreased fluorescence intensity [79].

**Figure 14:**
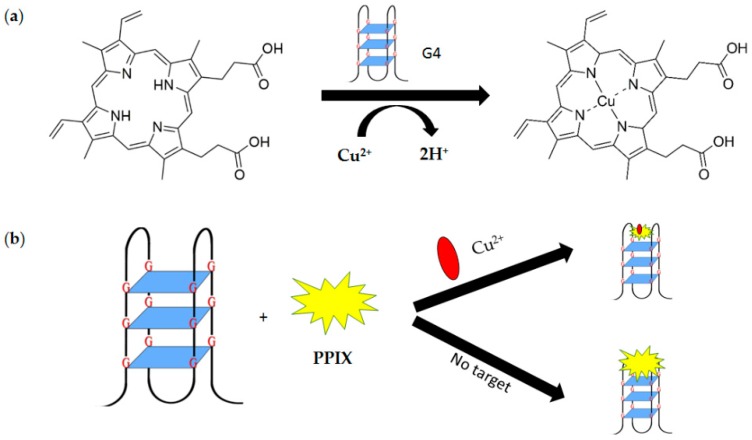
(**a**) Insertion of Cu^2+^ into PPIX (metalation) catalyzed by G4. (**b**) A schematic illustration of non-covalent fluorescence-based G-quadruplex sensing system utilizing PPIX for Cu^2+^ detection.

Uniquely, G4 is able to catalyze Cu^2+^ insertion into the PPIX (metalation) (see Figure 14a) quenching the fluorescence of the responding ligand to produce a decreased fluorescence intensity. However, a similar phenomenon is not reported when using other toxic metal ions [80]. High Cu^2+^ levels in vivo is claimed to be toxic especially when the levels are beyond cellular needs. This high level of Cu^2+^ can be associated with some serious neurodegenerative medical conditions such as Wilson disease, Menkes disease and Alzheimer’s disease [81]. Therefore, there is a need for a rapid and sensitive detection method of such metal ions. Taking advantage of this interaction of G4 in the presence of Cu^2+^, a sensitive G4-based probe for Cu^2+^ detection was developed [80]. PPIX acts as a fluorescent probe and binds to G4 in the absence of Cu^2+^ (see Figure 14b). 

The intensity of fluorescence increases sharply due to the binding. In the presence of Cu^2+^, PPIX-Cu complex forms and G4 binds to this complex. In this case, although the G4 still can bind to PPIX-Cu complex, the fluorescence intensity is much lower. This assay provides a reliable platform for effective detection with a low detection limit of 3.0 nM.

**Figure 15:**
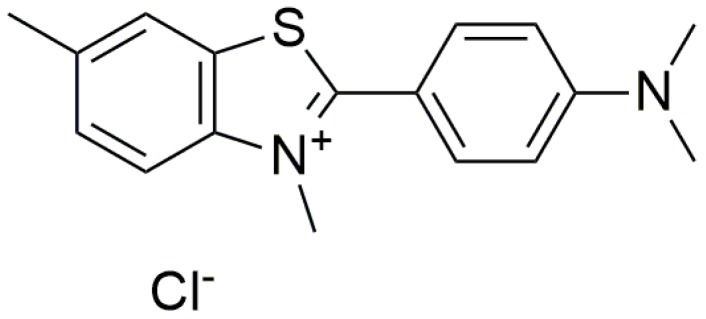
Chemical structure of thioflavin T (ThT).

In a later study, a detection system was developed surrounding the basis of the interaction between fluorophore thioflavin T (ThT) (see Figure 15) and G4. This involved the inducement of G4 flanking sites, which results in selective detection of the target sequences since ThT has a specific light-up fluorescence probe for G4 structures. ThT gives out very weak fluorescent signal in aqueous solution but a high fluorescent signal upon binding to G4. This structural specificity of ThT for G4 was taken advantage of to produce a sensitive assay. The formation of G4/ThT complex due to the hybridization of mutant DNA with the target DNA induced the G4 structure formation in the probe causing the ThT fluorophore to light up. This readout was used to elucidate the amount of a mutant ion in a target sequence with high sensitivity and specificity [82].

**Figure 16:**
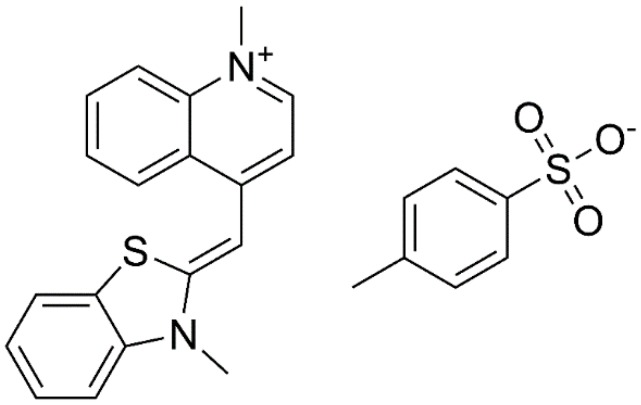
Chemical structures of thiazole orange (TO).

Thiazole orange (TO) (see Figure 16a) is one of the most commonly used fluorescent probes among the reported studies due to its high fluorescence quantum yield. However, TO lacks the ability to distinguish G4 from other types of DNA structures since it is a universal nucleic acid fluorescence dye [83]. It was reported that the introduction of hydrocarbon rings into the chromophore of thiazole orange alters the planarity of the chromophore and the binding affinity for G4 as well. With a suitable molecular framework to accommodate the TO chromophore, the altered TO possesses highly selective fluorescence response to G4 [84].

#### 3.2.2. Non-Covalent Fluorescence Aptamer-Based G-Quadruplex Sensing System

Aptamers are folded nucleic acids with a single-stranded structure (RNA or ssDNA), generally designed from 25 to 60 bases in length. The sequence variation allows the display of a large number of structural arrangements. This variability allows for sufficient structural diversity to generate the specific recognition cavities against a wide range of targets which includes small molecules, proteins, ions, whole cells and even entire bacteria and viruses [85,86]. Aptamers can be isolated from a large random-sequence library of either RNA or DNA by an evolution process called systemic evolution of ligands by exponential enrichment (SELEX). Through SELEX, a vast number of aptamers have been identified and it was reported that a number of aptamers underwent a conformational change within the G4 motif upon ligand binding [87]. DNA SELEX has mainly yielded more G4 aptamers with high affinities. G4 have a higher electrostatic potential per unit length compared to a duplex. When compared to a double helix, G4 have twice the negative charge density per unit length. Hence, G4 represent a good arrangement for differential interaction with small molecules or cationic proteins [88].

It is also assumed that in the absence of other possible interactions in RNA due to its free 2′-OH groups, the high stability of G4s are often required for DNA aptamers to adopt more complex non-canonical structures for ligand binding. Upon ligand binding, a nucleic acid aptamer converts from a non-quad or unstructured conformation into a G4 motif. This can then be effectively monitored through transduction into various outputs such as fluorescence and luminescence in a number of ways [87]. Aptamers are interesting alternative binders in sensory applications due to their simplicity, reusability and stability under a variety of environmental conditions. They are also cheaper to be synthesized and modified [89]. Aptamers also offer other advantages such as a lower molecular weight, reproducible synthesis, controllable labeling, flexible design, low immunogenicity and fast tissue penetration [90].

**Figure 17:**
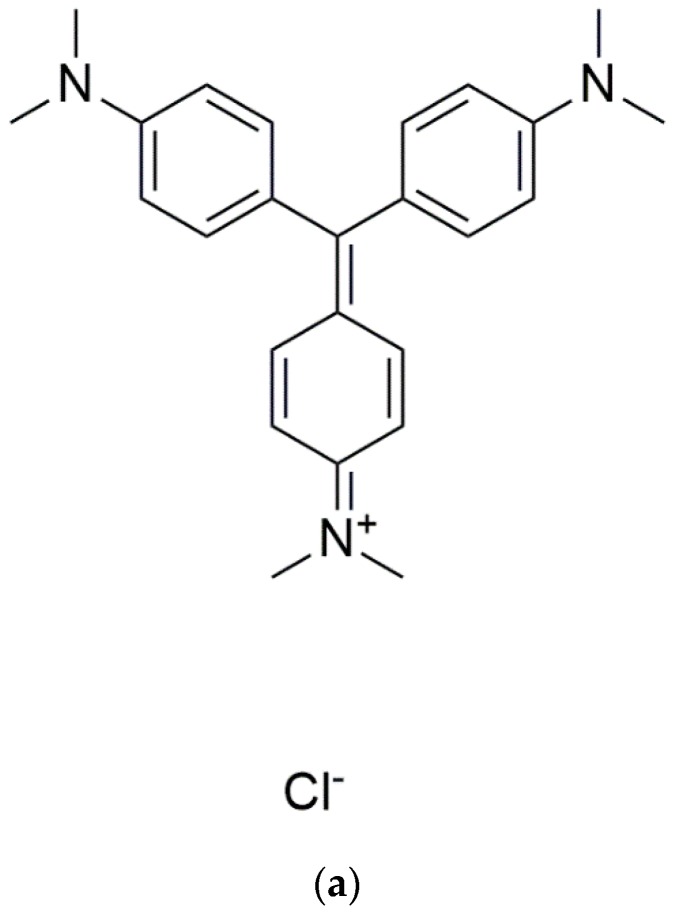

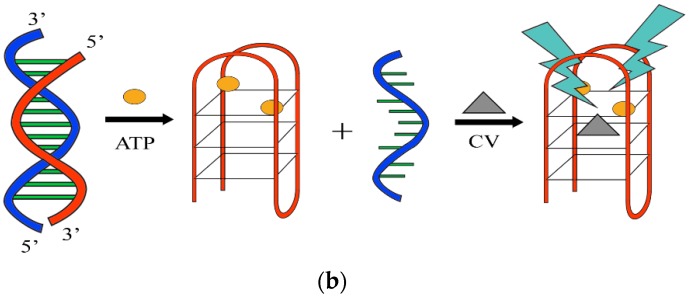
(**a**) Chemical structure of crystal violet. (**b**) Schematic representation of the aptamer-based fluorescent turn-on strategy for ATP detection using G-quadruplex probe crystal violet (CV). ATP promotes duplex dissociation and incudes formation of the aptamer-target G-quadruplex structure, which is detected by CV.

Since the conjugation of aptamers may lead to other unwanted issues and considering the unique feature of aptamers to adopt G4 structures upon binding to their cognate ligand, attempts have been made to develop a detection strategy devoid of aptamer modifications or without the use of enzymes. Various studies have been conducted utilizing non-covalent fluorescence on aptamer-based detection system. In one of these studies, a switch-on method for the detection of adenosine triphosphate (ATP) using aptamer based on a duplex-to-quadruplex conversion strategy was reported. Abnormal ATP levels from an overproduction of ATP by creatinine kinase has been associated with diseases like angiocardiopathy, making accurate detection of ATP a critical assessment for angiocardiopathy [91]. The ATP detection method applied a label-free fluorescence switch-on assay, utilizing crystal violet (CV) (see Figure 17a) to monitor the conversion of the ATP aptamer in the presence of ATP (see Figure 17b). It was also suggested that such a G4-based design could reduce the non-specific binding of the probe, while maintaining the simplicity and cost efficiency of label-free detection. In this system, the ATP aptamer and its complementary sequence were initially hybridized. In the absence of ATP, the binding of CV to the duplex DNA was weak resulting in a low emission of fluorescence signal. The duplex conformation was claimed to be in equilibrium with a small population of dissociated single strands allowing the ATP aptamer to form a G4 structure. Through population-shift mechanism, the presence of ATP will trigger the dissociation of the duplex and the formation of the aptamer-target complex as ATP only stabilizes the G4 structure of the aptamer. A strong interaction between the CV and the G4 was formed, producing a significant fluorescence response to ATP [92].

**Figure 18:**
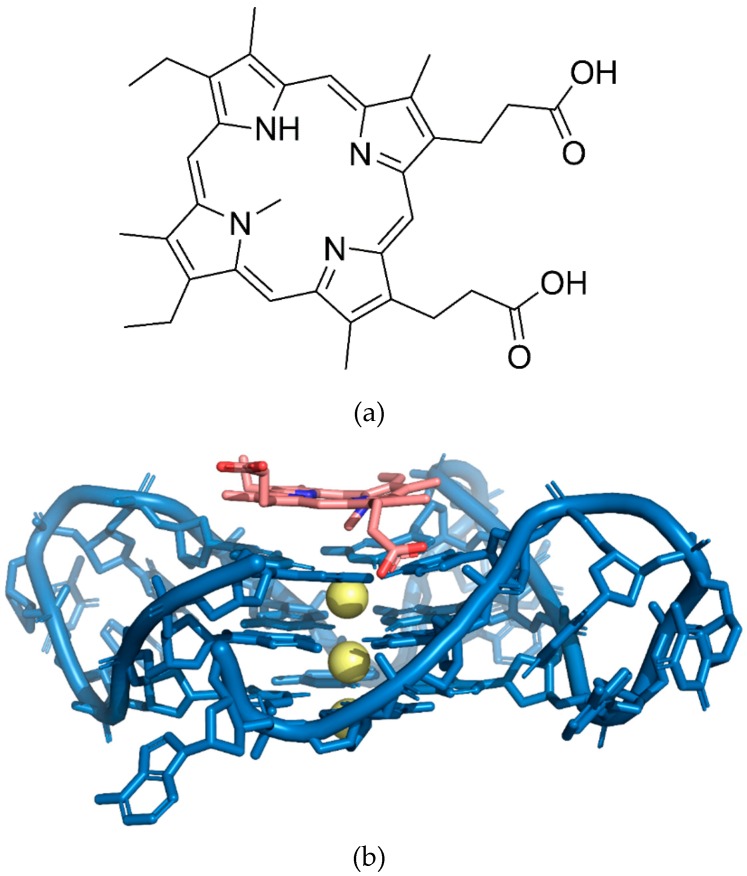
(**a**) Chemical structure of *N*-methylmesoporphyrin IX (NMM). (**b**) Structure of the complex of G-quadruplex and *N*-methylmesoporphyrin IX (NMM) (PDB: 4FXM).

A fluorescent dye, N-methylmesoporphyrin IX (NMM) (see Figure 18a) was used to detect the presence of thrombin [93]. The NMM was designed to bind to the formed G4 structure upon toehold strand displacement. The DNA formation will assemble to multiple G4 structures, emitting amplified fluorescent signals for improved sensitive detection. The system allowed for G4-forming sequences to be initially locked in the hairpin structures, meanwhile the initiation strand (IS) and the thrombin-binding aptamer sequence were partially hybridized. No free IS could be generated when the thrombin was absent and the G4-forming sequences remained engaged in the hairpin structures, avoiding the association of NMM. Low fluorescent emission could be expected in this case. The presence of thrombin promotes the release of IS as a result of the formation of the G4 aptamer-thrombin complex.

The liberated IS will then hybridize with the first hairpin strand and unfolds the hairpin structure, forming the toehold for strand displacement by the second hairpin strand. The second and unfolded hairpin strands will hybridize and the IS is then displaced via toehold strand displacement mechanism. The displaced IS will then hybridize again with the initial hairpin sequence to initiate the toehold strand displacement. This will finally result in a massive generation of dsDNA strands with both ends having active G4-forming sequences. The G4-forming sequences and NMM will associate to produce a sensitive monitoring system for thrombin by giving out remarkably intensified fluorescent emission [93]. Figure 18b shows the structural complex of a G4 and NMM.

### 3.3. Luminescence-Based G-Quadruplex Sensing System

#### 3.3.1. Conventional Luminescence-Based G-Quadruplex Sensing System

The characteristics of luminescent heavy metal complexes have gained large interest and attention especially towards the fabrication of light-emitting materials and numerous sensing applications due to its versatility, low cost, simplicity and high sensitivity. Their luminescence can be detected in profoundly fluorescent media as a result of their long emission life-time, with the utilization of time-resolved luminescence spectroscopy. Besides photophysical properties and interaction with biomolecules, they require no complicated synthetic procedures and are readily available for fine-tuning. In addition, self-quenching can be prevented as they possess remarkable stokes shifts [94]. As a consequence, the potential of several luminescent heavy metal complexes such as ruthenium (II), iridium (III), and platinum (II), as molecular light switches for nucleic acids including G4 DNA have been widely studied.

**Figure 19:**
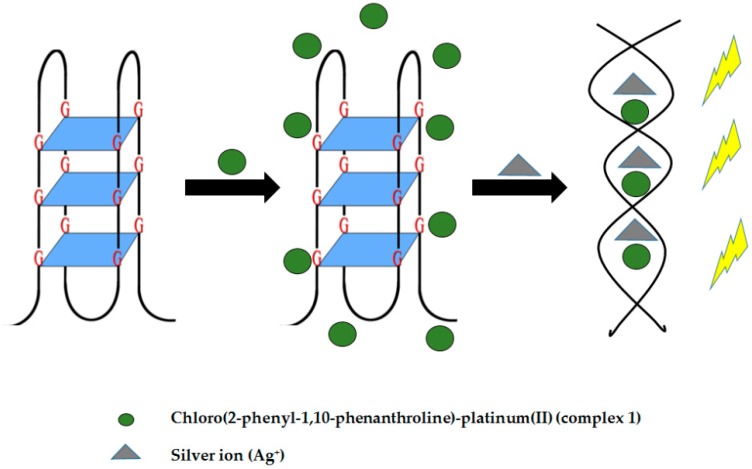
A schematic illustration of luminescence-based G-quadruplex assay for aqueous silver ions detection. (**a**)The initial structure of G-quadruplex. (**b**) The poor interaction of G-quadruplex and complex 1. (**c**) The silver ions induce the G4-to-duplex conformational change, allowing the intercalation of complex 1 and resulting in the emission of intense phosphorescence.

Silver ions develop cytotoxicity in humans [95] and are associated to a number of medical complications including organ failure [96]. This highlights the importance of silver ion level determination in the body. To carry out silver (I) ion detection in aqueous solution, a luminescence-based G4 sensing system was developed utilizing chloro(2-phenyl-1,10-phenanthroline)-platinum(II) (complex 1). As Ag^+^ ions are able to destabilize the G4 structure [28], G4 structures can undergo conformational change from G4 to a duplex structure. The formed duplex structure allows the intercalation of complex 1, producing a high emission intensity (see Figure 19). However, low emission intensity is observed in the absence of Ag^+^ owing to the weak interaction of complex 1 with G4. Upon addition of various metal ions other than silver, the emission intensity only increases slightly suggesting the selectivity of the system for Ag^+^ ions [97].

#### 3.3.2. Luminescence Aptamer-Based G-Quadruplex Sensing System

Human neutrophil elastase (HNE) is a type of serine protease that degrades a variety of functional and structural proteins, which includes collagen, proteoglycan, fibronectin and laminin. However, the destruction of normal tissues may occur if the HNE is overproduced. This condition has been associated with the development of various autoimmune disorders. For this reason, an accurate detection platform for the diagnosis of HNE-related diseases is crucial. A selective and sensitive switch-on assay to detect sub-nanomolar HNE in homogeneous solution was reported. This assay was conducted using a label-free aptamer-based strategy utilizing a G4-selective luminescent iridium (III) complex [Ir(ppy)2(dpp)]^+^ (where ppy = 2-phenylpyridine and dpp = 2,9-diphenyl-1,10-phenanthroline) as a signal-transducing element [98]. In this system, either in an aqueous solution or in the presence of dsDNA containing the HNE aptamer, Complex 1, [Ir(ppy)2(dpp)]^+^, is weakly emissive. The dissociation of the DNA duplex was induced with the addition of HNE. The HNE aptamer is then released and will subsequently fold into a G4 structure. An enhanced luminescence response that could be readily viewed under UV-illumination was produced due to the strong interaction of Complex 1 with the HNE-aptamer. Therefore, a linear relationship was observed between the HNE concentration and the luminescence intensity of Complex 1. The range and limit of detection of this system was of the same standard as the commercial ELISAs. Although the MB-based approach was reported to be more sensitive than this assay [99], this assay is considered useful due to the lower cost, simplicity and eliminates the involvement of the covalent labeling of oligonucleotides [98].

**Figure 20:**
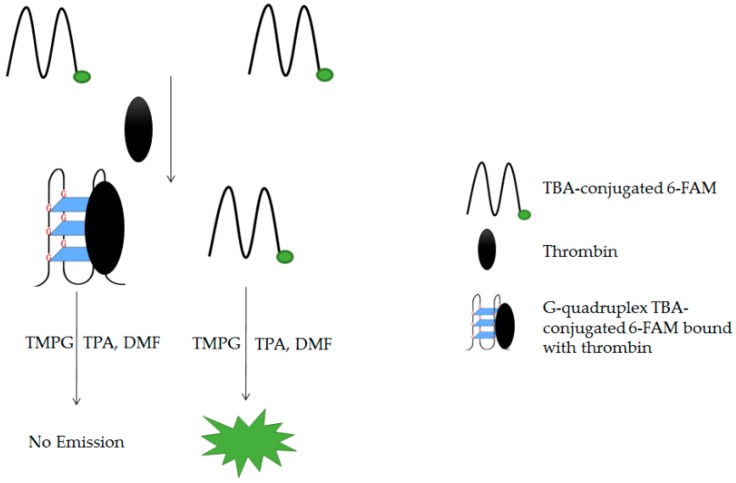
Schematic illustration of G-quadruplex-TBA aptasensor with guanine chemiluminescence detection principle.

Chemiluminescence is luminescence where energy is supplied by chemical reactions. In a reported study, a thrombin aptamer was used to develop a cost effective sensitive aptasensor with guanine chemiluminescence detection [100]. This system is capable of quantifying thrombin in human serum without the need of tedious procedures and expensive nanoparticles. In the presence of tetra-*n*-propylammonium hydroxide (TPA), guanine of G4 TBA-conjugated carboxyfluorescein (6-FAM) that is bound with thrombin would not react with 3,4,5-trimethoxylphenylglyoxal (TMPG) (see Figure 20). Guanines of free TBA and TBA-conjugated 6-FAM will rapidly react with TMPG by immobilization on the surface of graphene oxide to emit light. As a result of the formation of G4 TBA-conjugated 6-FAM bound with thrombin in a sample, the brightness of guanine chemiluminescence was quenched. The reaction between guanines of TBA and TMPG in the presence of TPA formed an intermediate of high energy that was capable of emitting dim light by itself. Consequently, based on the principle of chemiluminescence energy transfer (CRET), energy was transferred to 6-FAM to emit a bright light. Using the technology reported in this study, various types of G4 DNA aptasensors capable of specifically sensing a target molecule could be developed [100]. These target molecules may include ATP, HIV, ochratoxin, potassium ions, and thrombin.

### 3.4. G-Quadruplex-Based Higher Order Sensor and Actuator Approaches

As G4s can form high affinity interactions with otherwise separate oligonucleotide strands, other high molecular weight approaches in sensing and actuation have been explored. For instance, the potassium-dependent contraction of a long human telomeric repeat sequence was observed by surface plasmon resonance (SPR) that could measure the change of mass within its evanescent field [101]. Technically, fluorescence energy transfer (FRET) can be used to directly measure the structural change but with the disadvantage of using labels, or by direct probing using atomic force microscopy. In contrast, using this principle of SPR, an on-line and direct measurement of structural change can be performed without the need of labels. The changes in refractive index resulting from the binding events or in the structure of biomolecules such as RNA are able to be measured too [101]. Thus, it may be possible to detect interactions that influence the formation of G4s by mere mechanical means. At the same time, it is conceivable that such a higher order conformational change can be employed as an actuator responding to a specific trigger as well.

Alternatively, the principle shown in Figure 5 has been expanded to generate high molecular weight complexes based on hybridization chain reaction (HCR) [102] that has more recently been devised into a label free method [103]. Unlike the strategy of splitting-DNAzyme, locking the intact G4 sequence into hairpin probes of HCR was proposed as an attractive nucleic acid detection system which is protein-free, isothermal and capable of self-amplifying [102]. In this approach, the G4 was closed one-third in the stem of one of the GQ-HCR hairpin probes and two-thirds in the loop. The GQ-HCR probes stayed as inactive meta-stable hairpin structures in the absence of the target molecule resulting in an inert G4. As HCR could be initiated by cross-opening of G4 probes, when the GQ-HCR probes comes across the target molecule the closed G4 could be freed.

Even higher order structures have been selectively assembled or switched based on G4 formation. DNA Origami technology shows some prospect in terms of new applications for G4s. A FRET-based sensor system using a triangular scaffold was shown to be highly responsive to potassium ion concentrations [104]. This hints at the potential application of G4s for conditional release or capture of molecules when entering a cell that normally has higher potassium concentrations than the outside medium. Yet complete macromolecular folding blocks can rearrange in response to analytes to gain an enzymatic function. A potassium-dependent switch of such blocks aptly termed “dominoes” has been demonstrated most recently by the research group of Willner [105]. Given that such rearrangements can be achieved depending on the presence of specific analytes, it may be possible to detect the formation of a regular higher order structure in liquid crystals by the naked eye that can be applied in completely novel bottom-up sensor concepts. This highlights the tremendous freedom one can be accorded when utilizing G4 systems for sensing and actuation applications.

The ability for G4 structures to be adapted for different sensing platforms allows great flexibility in sensor designs. Table 2 summarizes the different sensing platform that can be designed with the utilization of G4 structures. The many different G4 derived sensing platforms highlights the usefulness to apply G4 as an alternative sensing platform for diagnostic applications.

## 4. Conclusions

The versatility of the G4 motif has created a prominent impact towards the field of sensing. The number of potential applications of G4 has vastly expanded with the increasing number of studies on G4. Compared with antibodies or organic chemosensors, DNA oligonucleotides possess various remarkable advantages which includes high thermostability and solubility, ease of production and modification as well as rich structural polymorphism in the presence of particular targets, and can be responsive. In particular, the systems that have been described in this review focused on ‘label-free’ strategies. These label-free approaches have recently gained popularity due to its simplicity and does not require time-consuming labelling and immobilization steps.

The discovery of G4 DNA has spawned an entire discipline and new perception of DNA. The various applications of G4 DNA for the development of different sensing systems highlights the robustness of G4 to function as a reporter system in future sensor platforms. It is conceivable that G4 reporter systems will play a more active role in sensor developments. Recent combinations of G4s with DNA Origami may lead to the discovery of entirely novel sensor concepts. In conclusion, G4 based reporter systems could serve as an attractive alternative to existing reporter systems available to date.

## Figures and Tables

**Table 1:** Thermal denaturation profile of different conformations of G4.

Conformation	λ (nm)			
	243	260	275	295
Parallel	+	−	−	−
Antiparallel	+	−/+	+	−
Hybrid	+	+	+	−

(+) Hyperchromism and (−) hypochromism observed upon denaturing/heating the structure.

**Table 2:** Characteristics of different G4-based detection platforms.

Sensing System	Target Molecules	Facilitating Ligands	Advantages	Disadvantages	Limit of Detection	References
G4/Hemin-basedDNAzyme	ssDNA (SNP)	Hemin	Simple, fast analysis and low cost Easy to prepare and purify as compared with natural protein enzymes Requires no expensive and sophisticated instrumentation No tedious covalent labelling Instant and visible colour change High sensitivity and selectivity More desirable because in some cases, organic dyes undergo photobleaching and possess poor aqueous solubility In contrast to nanomaterials-based peroxidase mimics, G4/hemin DNAzymes are commonly water-soluble	-	0.95 nM	[56]
	Sodium ions				0.6 µM	[58]
	Hydrogen sulfide (H_2_S)	Hemin, Ag^+^ and Tb^3+^ (as substitute for K^+^)	Exhibits higher catalytic activity than G4/hemin-based DNAzyme induced by other commonly-used cations High selectivity as it is not affected by the presence of other anions Can be applied on serum sample without affecting the precision	When the concentration of H_2_S is higher than a certain level, Ag^+^ would not be able to transform into AgS causing the absorbance of ABTS-H_2_O_2_ solution to remain the same.	13 nM	[29]
DNAzyme-based colorimetric split G4	ssDNA (rifampin-resistant *M.tb*)	Hemin	Split probes exhibit excellent selectivity against nucleic acid	Probe with multiple GGG repeats could form an intramolecular G4 structure and produce a significant background signal (Problem was solved by adding a sequence to its 5′-end that is partially complementary to the 3′-end ) Probe could form a stable secondary structure and result in a low signal readout	-	[106]
DNAzyme-based colorimetric G4/Hemin Molecular Beacon	nrDNA ITS		Does not involve fluorophore/quencher complex labeling as conventional molecular beacons do Does not involve chemical modification DNAzyme MB can act as a primer for polymerization Provide sequence specific recognition in a straightforward fashion DNAzyme MB is able to exhibit SDA effect, act as target DNA recognition element and generate signal	-	3.1 × 10^−10^ mol·L^−1^	[70]
	p53 DNA			-	25 fM	[71]
Conventional non-covalent fluorescence G4	DNA (base number of DNA)	Protoporyphrin IX (PPIX)	Sensitive to single-nucleotide addition Binding affinity between PPIX and G4 is largely dependent on the integrity of G4 structure Does not involve probe fluorophore labeling and modification Is not interfered by other metal ions Exhibit high specificity and sensitivity, enabling efficient detection of target mutant DNA	The fluorescence intensity changes only little when the fluorescence has reached a plateau at a certain concentration.	-	[79]
	Copper (II) ion				3.0 nM	[80]
	DNA (EGFR exon 19 deletion mutant)	Thioflavin T (ThT)			2.3 nM	[82]
Non-covalent fluorescence aptamer-based G4	ATP	Crystal violet (CV)	Reduce the non-specific binding of the probe, while maintaining the simplicity and cost efficiency of label-free detection. The interfering NaCl or glucose deso not induce significant fluorescent changes in the system Potential to be applied for the detection of ATP in real samples. Eliminates the requirement for fluorescent labeling of DNA aptamer while the robustness is still maintained due to the selective CV–G4 interaction.	-	5 µM	[92]
	Thrombin	*N*-Methyl mesoporphyrin (IX) (NMM)	Enzyme-free Non-labeled, exhibits better binding affinity since aptamer modification may alter their binding affinity Able to assay thrombin in real samples High selectivity which is associated with the highly specific binding between the target thrombin and the corresponding aptamers.	-	5 pM	[93]
Conventional luminescence-based G4	Silver ion	Chloro(2-phenyl-1,10-phenanthroline)-platinum(II)	Simple and cost-effective Sensitive and highly selective Its selectivity is comparable to oligonucleotide-based systems Possesses excellent solubility in aqueous solution The range and limit of detection of this are of the same standard as the commercial ELISAs. Eliminates the involvement of the covalent labeling of oligonucleotides Excellent sensitivity compared to molecular beacon-based methods	-	20 nM	[97]
Luminescence aptamer-based G4	Enzyme	Iridium(III) complex[Ir(ppy)_2_(dpp)]^+^		MB-based approach was reported to be more sensitive than this assay	-	[98]
	Thrombin	3,4,5-trimethoxy-phenylglyoxal (TMPG)	Simple, rapid and cost-effective Highly sensitive guanine chemiluminescence detection without expensive and intractable nanoparticles including magnetic Fe_3_O_4_GO nanoparticles Involves no complicated procedures Capable of quantifying thrombin in human serum	-	12.3 nM	[100]
